# Papillary Neoplasms of the Gallbladder and Extrahepatic Bile Ducts: A Report of Two Cases With Associated Invasive Carcinoma

**DOI:** 10.7759/cureus.58415

**Published:** 2024-04-16

**Authors:** Nadir Miry, Younesse Najioui, Anass Haloui, Nassira Karich, Amal Bennani

**Affiliations:** 1 Pathology Department, Mohammed VI University Hospital, Oujda, MAR

**Keywords:** bile duct pre-malignant lesion, gallbladder pre-malignant lesion, bile duct neoplasm, gallbladder neoplasm, intraductal papillary neoplasm, intracholecystic papillary neoplasm

## Abstract

Intracholecystic papillary neoplasm (ICPN) of the gallbladder is a macroscopically visible premalignant lesion protruding into the gallbladder lumen, with infrequent association with invasive adenocarcinoma. Intraductal papillary neoplasm of the bile ducts (IPNB) is a non-invasive lesion characterized by intraductal papillary or villous architecture. Both ICPN and IPNB are rare findings in the gallbladder and biliary tract pathology. Diagnosis relies on clinical manifestations, imaging techniques, and comprehensive histological examination. Here, we present two cases: a 63-year-old male with mild abdominal pain found to have a gallbladder mass, diagnosed histologically as ICPN with associated invasive carcinoma; and a 65-year-old female with chronic jaundice and a large tumor mass in the common bile duct, histologically diagnosed as IPNB with associated invasive carcinoma. These cases highlight the importance of a careful and thorough microscopic examination to rule out differential diagnoses and to reveal any potential invasive carcinoma associated with these uncommon lesions.

## Introduction

The most recent WHO classification (5th edition) recognizes pyloric gland adenoma, biliary intraepithelial neoplasia, mucinous cystic neoplasm, intracholecystic papillary neoplasm (ICPN), and intraductal papillary neoplasm of the bile ducts (IPNB) as benign epithelial neoplasms and precursor lesions of the gallbladder and extrahepatic bile ducts [1]. ICPN, a recently described preinvasive neoplasm, is incidentally identified in less than 4% of cholecystectomy specimens [2]. The neoplastic cells exhibit varying dysplastic grades and display diverse phenotypes, including gastric, intestinal, oncocytic, and pancreatobiliary subtypes [3]. Lesions with invasive carcinoma components are recognized as ICPN with associated invasive carcinoma [2].

On the other hand, IPNB is a rare preinvasive lesion originating from bile epithelium, affecting any segment of the biliary tract. It is defined as a grossly visible, premalignant neoplasm, consisting of intraluminal papillary and villous structures [4]. It can be categorized into four subtypes, including intestinal, pancreatobiliary, gastric, and oncocytic subtypes. In the presence of invasive features, the term IPNB with associated invasive carcinoma is recommended. A two-tier classification of IPNB is established based on the degree of architectural complexity, with type one exhibiting more regular morphology and a more favorable postoperative outcome. It was documented that approximately half of IPNBs demonstrate simultaneous stromal invasion [5], thus emphasizing the importance of a thorough sampling and a careful microscopic evaluation of such lesions to exclude any potential associated invasive carcinoma.

## Case presentation

Case 1

**Figure 1:**
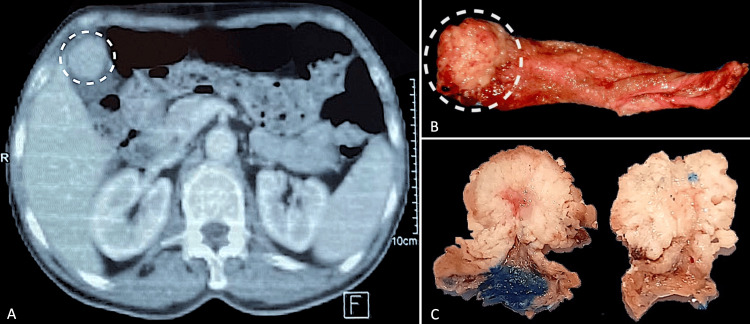
Axial abdominal CT scan showing a well-circumscribed solid lesion (circle), attached to the anterior wall of the gallbladder fundus (A). Gross examination of the cholecystectomy specimen reveals a bulging, intra-luminal lesion (circle) (B); cross-section of the lesion shows a polypoid formation with no macroscopically evident wall invasion (C).

We present in the first case a 63-year-old male patient, without any significant medical history, who experienced chronic mild pain in the right hypochondrium for the past month. No jaundice or other general symptoms were reported. Abdominal examination revealed a slight tenderness at the right hypochondrium without perceptible abdominal mass or hepatomegaly; the rest of the physical examination didn’t show any abnormalities. Ultrasound imaging revealed a mass lesion in a non-distended gallbladder. The CT scan shows a relatively well-circumscribed mass measuring 27x26mm at the fundic region of the gallbladder (Figure 1A), and displaying a heterogeneous contrast enhancement. No wall thickening, gallbladder distention, or gallstones were identified. Blood tests showed no hepatic markers disruption. Following a cholecystectomy, a macroscopic examination revealed an intraluminal polypoid lesion with a villous appearance, located in the gallbladder fundus and measuring 25x25x17mm (Figures 1B, 1C).

**Figure 2:**
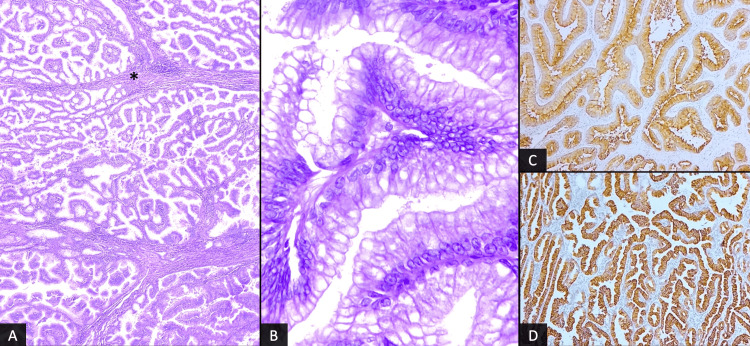
Photomicrographs of the lesion show multiple papillary structures with fine fibro-vascular cores (*) (A: H&E, x40). At higher magnification, the epithelial lining is made of simple, columnar cells with clear to eosinophilic cytoplasm and enlarged nuclei with distinct nucleoli (B: H&E, x400). Immunohistochemical staining shows a diffuse positivity of tumor cells for both EMA (C) and Ck7 (D).

Histologically, the tumor consisted of an intraluminal proliferation of tubular and papillary structures with fine fibrovascular cores and minimal intervening stroma (Figure 2A), lined by cuboidal to columnar cells with abundant mucinous cytoplasm (Figure 2B). A complex architecture with cribriform and solid growth patterns invading the stroma was observed in the lamina propria of the gallbladder. On immunohistochemical examination, tumor cells showed a diffuse and strong expression of EMA (Figure 2C) and Ck7 (Figure 2D), with only focal expression of MUC5, and negative reaction for CDX2. Therefore, the tumor was diagnosed as ICPN with associated invasive carcinoma, staged as pT1a.

Case 2

**Figure 3:**
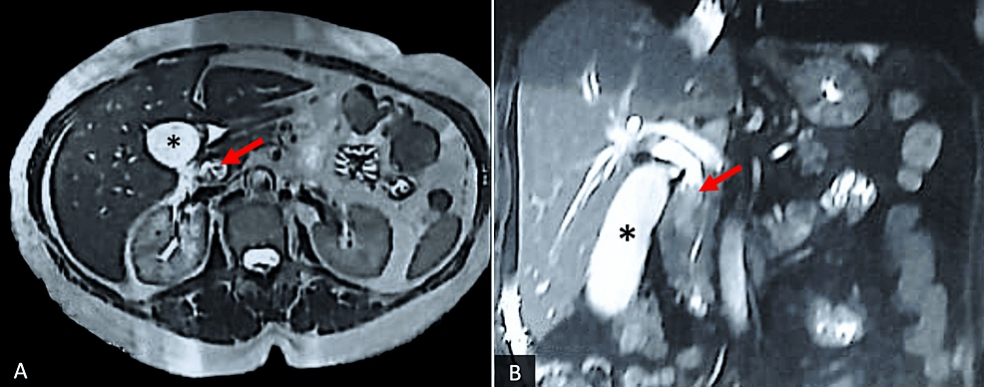
Axial abdominal MR T2-weighted imaging shows a dilated common bile duct (arrow), adjacent to the gallbladder (*) (A). Coronal MR T2-weighted imaging shows the gallbladder (*) with a thickened and dilated common bile duct upstream of a solid obstacle (arrow) (B).

The second case involved a 65-year-old female patient with a history of diabetes and arterial hypertension. She had been experiencing chronic jaundice for three weeks before seeking consultation, no fever or abdominal pain were reported. The physical examination did not reveal any significant abnormalities. However, laboratory findings indicated elevated levels of liver enzymes, including high glutamic-pyruvic transaminase (GPT), glutamic-oxaloacetic transaminase (GOT), alkaline phosphatase, and gamma-GT. Tumor markers revealed an increased level of CA19.9 (Carbohydrate antigen 19.9) at 170 UI/mL. The CT scan revealed a dilated common bile duct measuring 12mm, with an intra-biliary tumor mass. The mass in the common bile duct exhibited a low signal on T1-weighted imaging (Figures 3A, 3B) and a high signal on T2-weighted imaging, with no evident signs of invasion.

**Figure 4:**
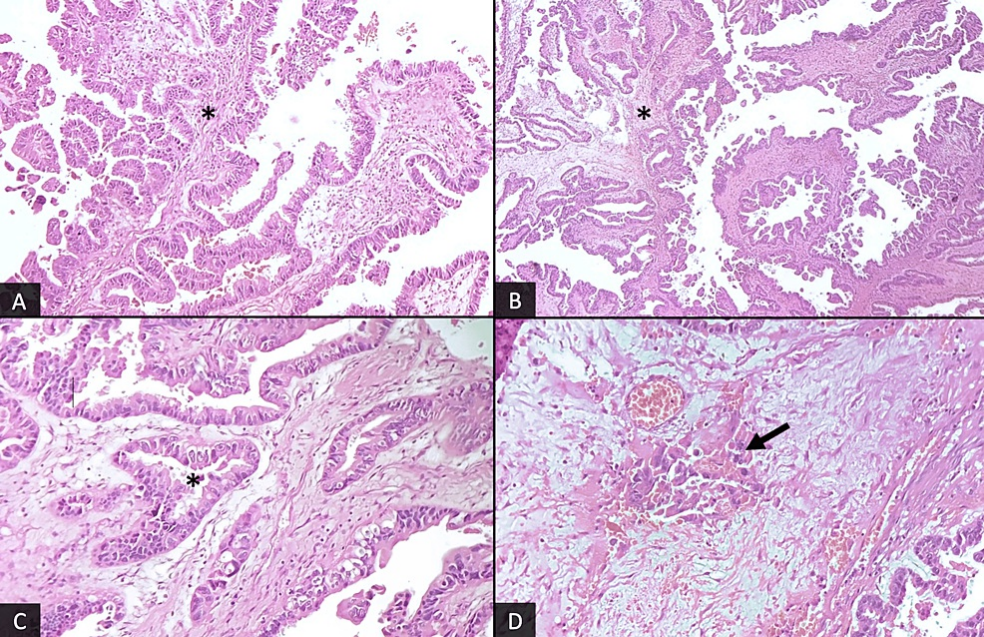
Photomicrographs of the resected specimen show a papillary neoplasm with complex tubulo-villous architecture, containing both papillae (*) (A, B: H&E, x40), and tubular structures (*) (C: H&E, x200). A focus of invasion can be noted (arrow) (D: H&E, x200).

The patient underwent a resection of the common bile duct, along with lymph node dissection. Microscopic examination revealed an intra-biliary tubulo-papillary proliferation composed of fibrovascular cores (Figures 4A, 4B) lined by columnar cells with enlarged nuclei and often prominent nucleoli and exhibiting epithelial stratification. The cytoplasm was abundant and eosinophilic (Figure 4C). Small foci of invasion were readily identified, characterized by irregular glands infiltrating the lamina propria with no perineural or vascular invasion (Figure 4D).

Finally, the patient was diagnosed with intraductal papillary neoplasm of the common bile duct with associated invasive carcinoma.

## Discussion

ICPNs are papillary lesions involving the gallbladder, characterized as macroscopically visible, mass-forming, and noninvasive epithelial neoplasms originating from the gallbladder mucosa and protruding into the lumen [6]. These lesions are analogous to IPNB and intraductal papillary mucinous neoplasm (IPMN) of the pancreas, distinct preinvasive lesions of the pancreato-biliary tract [7]. The average age of individuals diagnosed with ICPN typically falls around 60 years old. A notable female predominance is reported, with a sex ratio of about 3 to 6:1 [8]. Approximately half of patients with ICPN experience pain in the right upper quadrant of the abdomen, while the remaining patients are asymptomatic. From a macroscopic perspective, the median tumor size is approximately 2cm, occasionally presenting as a multifocal lesion [6]. Typically, when there is no obstruction, particularly in the cystic duct, laboratory results tend to be normal. On imaging, the gallbladder commonly exhibits a thickening of its wall and the presence of an intra-luminal mass [9].

Histologically, ICPN shows tubular and papillary structures with fine fibrovascular cores and minimal intervening stroma [2]. It is imperative to differentiate ICPN from other pre-invasive lesions of the biliary tract, such as Biliary Intraepithelial Neoplasms (BilIN), which are solely microscopically identifiable and often present as flat or micropapillary formations. Distinct from ICPN, Pyloric Gland Adenoma (PGN), a recently recognized and distinct entity, characterized by densely packed pyloric-like or Brunner-like glands devoid of intervening papillae or villous structures, the epithelial cells show abundant apical mucinous cytoplasm that pushes the nuclei to the periphery. Invasive carcinoma of the gallbladder with considerable intraluminal papillary component should be distinguished from ICPN with associated gallbladder wall invasion.

Two types of ICPN are described based on architectural irregularities, with type 2 ICPNs characterized by the presence of complex architectural features such as cribriform, solid, and compact tubular structures, along with variably sized cystic changes, and frequent association to high-grade dysplasia and invasive adenocarcinoma [2]. Type 1 ICPNs, on the other hand, exhibit a relatively regular architectural pattern with a more favorable outcome. Furthermore, four morphologic subtypes of ICPN have been identified, including pancreato-biliary, intestinal, gastric, and oncocytic subtypes [3]. The most prevalent morphologic subtype of ICPN is the pancreato-biliary subtype, characterized by cuboidal epithelium displaying clear to eosinophilic cytoplasm, enlarged nuclei, and prominent nucleoli. The intestinal subtype shares similarities with colonic adenomas, featuring tall columnar cells exhibiting pseudostratified nuclei and basophilic cytoplasm. In the oncocytic subtype, arborizing papillae are observed, lined by multiple layers of epithelial cells with abundant eosinophilic granular cytoplasm and single prominent nucleoli. The gastric subtype can manifest as a foveolar type, resembling gastric foveolar epithelium with elongated glandular structures lined by columnar cells with mucinous cytoplasm. Alternatively, it may present as a pyloric type, composed of small tubular glands lined by uniform cuboidal cells with round nuclei and a moderate amount of cytoplasm, as mentioned in the 5th WHO classification. 

When a particular subtype dominates and constitutes more than 75% of the tumor, it is designated as a mono-subtype. If the tumor exhibits substantial regions featuring two or more subtypes, it is appropriately categorized as a mixed-subtype ICPN [2]. The gallbladder lesion in our case showed a proliferation of both tubular and papillary structures, devoid of any gland-like structures, with epithelial cells featuring a cuboidal to columnar shape without pseudo-stratification. Additionally, the nuclei were mostly round to ovoid, with abundant eosinophilic cytoplasm containing a moderate amount of mucin, which was consistent with a predominant pancreato-biliary morphology. Immunohistochemical staining serves as a valuable tool for subtype identification, with gastric foveolar and pyloric ICPNs exhibiting positive staining for MUC5AC and MUC6, respectively. Intestinal ICPNs typically display positive staining for CK20, CDX2, and MUC2. The oncocytic subtype demonstrates diffuse positivity for MUC1 (EMA), while the pancreato-biliary type exhibits positivity for CK7 and MUC1 [6]. Our first case exhibited diffuse staining for EMA and Ck7, thus confirming the pancreato-biliary phenotype.

ICPNs are precursor lesions for gallbladder adenocarcinomas that could be associated with low-grade dysplasia, high-grade dysplasia, or occasionally, invasive adenocarcinoma [9]. Therefore, a meticulous pathological analysis is crucial for accurately diagnosing these lesions and assessing their prognosis. In our case, only a limited invasion was noted, as a focus of complex architecture and more pronounced atypia, which prompted the diagnosis of ICPN with associated invasive carcinoma. 

IPNB is a benign and pre-malignant lesion that could affect both intra and extra-hepatic biliary tracts. It occurs mostly in males in their sixth decade and is typically responsible for abdominal discomfort and jaundice. The median tumor size varies between 2.2cm and 6cm, including cystic forms [10]. Radiological assessments typically show an intraluminal papillary lesion with bile duct dilation [11]. Macroscopically, it consists of a papillary proliferation with fine fibrovascular cores containing inflammatory cells and edema, covered by biliary epithelial cells, and possibly admixed tubular or glandular components. Epithelium of IPNBs shows intestinal, biliary, oncocytic, or gastric-type differentiation based on cytological appearance and immunophenotype, with frequent association of at least two types of epithelia [11].

IPNBs are divided into two categories based on their similarity to pancreatic IPMN. Type 1 IPNBs share close morphological similarities to IPMN and are more frequently intrahepatic. Whereas type 2 is more prevalent in the extrahepatic bile ducts and is associated with a less favorable prognosis. It should be distinguished from micropapillary biliary intraepithelial neoplasia (BilIN) and intraductal tubulo-papillary neoplasm (ITPN). The extrahepatic IPNB is known to be more aggressive, with more frequent high-grade dysplasia, architectural complexity, and stromal invasion often as tubular adenocarcinoma, compared to its intrahepatic counterpart [12]. In the second case, the intra-ductal lesion showed a proliferation of tubules and papillae with columnar epithelial cells featuring large nuclei and often prominent nucleoli, with abundant eosinophilic cytoplasm, compatible with a pancreato-biliary morphology; small foci of invasion were also seen.

MR imaging findings such as large tumor size (>2.5cm), the presence of multiple lesions, or a thickening of the bile duct wall could indicate the presence of an invasive component. However, in the absence of clear invasion on imaging, histomorphological examination remains the most reliable approach to confirm the presence of any potential invasion [11]. In the presence of invasion, the WHO classification recommends classifying the lesion as IPNB with associated invasive carcinoma. Invasive adenocarcinoma derived from IPNB has shown a more favorable prognosis than conventional cholangiocarcinomas. In both ICPN and IPNB, a complete resection of the lesion is recommended when clinical symptoms and radiological images are suggestive [9,11].

## Conclusions

ICPN and IPNB are part of the same spectrum of lesions that can affect both the biliary and pancreatic tracts, yet they remain relatively rarely reported in the literature. These lesions exhibit numerous clinical and histological similarities. While their association with invasive lesions is not uncommon, they tend to display less aggressive behavior compared to invasive neoplasms originating from other pre-neoplastic lesions in the gallbladder and biliary tract. A thorough pathological examination of these lesions remains a major step for identifying the nature of the lesion and the presence of invasion that could influence the patient’s prognosis and survival.
